# The Role of Hypoxia-inducible Factor-1 in Bladder Cancer

**DOI:** 10.2174/1566524023666230720163448

**Published:** 2023-08-24

**Authors:** Jiagui Chai, Sifan Yin, Wenbo Feng, Tao Zhang, Changxing Ke

**Affiliations:** 1 Department of Urology, the Second Affiliated Hospital of Kunming Medical University, Kunming, 650106, China;; 2 Yunnan Institute of Urology, Kunming, 650106, China

**Keywords:** HIF-1, bladder cancer, glucose metabolism, angiogenesis, proliferation, metastasis, drug resistance, treatment

## Abstract

Bladder cancer (BC) is one of the most common malignant tumors worldwide and poses a significant hazard to human health. During the development of BC, hypoxia plays a crucial role. Hypoxia-inducible factor (HIF) is a key transcription factor for hypoxic adaptation, which regulates the transcription of various genes, including inflammation, angiogenesis, and glycolytic metabolism. Recent studies have shown the precise role of HIF in various biological behaviors of BC. More importantly, a new antitumor medication targeting HIF-2 has been used to treat renal cancer. However, therapies targeting HIF-1 in BC have not yet been developed. In this review, we discussed how HIF-1 is expressed and affects the growth, metastasis, and angiogenesis of BC. At the same time, we investigated several HIF-1 inhibitors that provide new perspectives for targeting HIF-1.

## 1 INTRODUCTION

In malignant tumors, growth is accelerated due to rapid angiogenesis and/or proliferation [1]. However, the unstable blood flow from tumor neovascularization and the substantial oxygen consumption due to the rapid proliferation of cells will result in an imbalance between oxygen supply and consumption, which will cause hypoxia in the tumor microenvironment (TME) [2]. Accordingly, tumor cells will trigger a series of adaptive responses for tumor adaptation to the hypoxic environment, such as increased synthesis of specific proteins [3].

Recently, increasing studies have revealed that the hypoxia-inducible factor-1 (HIF-1), a member of the HIF family and consisting of HIF-1α and HIF-1β subunits, is a critical factor in tumor adaptation to hypoxia [4-6] and its synthesis is increased under hypoxia Fig. (**1**).

Specifically, HIF-1α is hydroxylated in the presence of prolyl hydroxylase (PHD) and then identified by von Hippel-Lindau (VHL), leading to the normoxic degradation of HIF-1α [7, 8]. When cells are subjected to hypoxia, PHD will become inactive, which prevents HIF-1α degradation and indirectly promotes HIF-1α synthesis [9]. At the same time, cell hypoxia will produce reactive oxygen species (ROS), which can directly or indirectly promote HIF-1α synthesis [4].

**Fig. (1):**
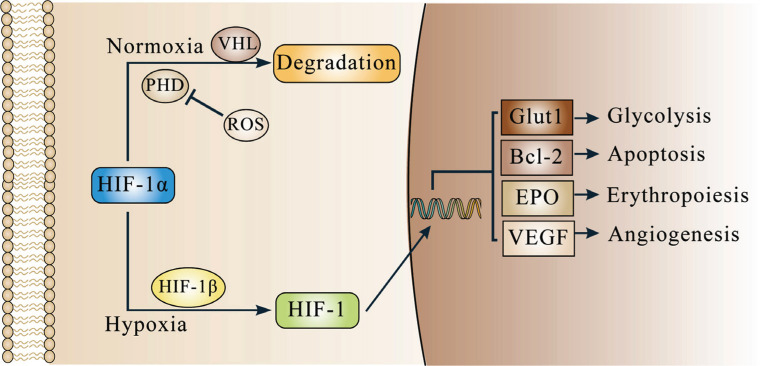
HIF-1α Synthesis and action mechanism at various oxygen levels. Under normoxia, HIF-1α is degraded. HIF-1α, on the other hand, is not broken down under hypoxia and enters the nucleus to promote gene transcription.

When HIF-1α is synthesized, it binds to HIF-1β to form the HIF-1 complex and enters the nucleus. HIF-1 then attaches to the hypoxia-response element (HRE) and promotes downstream gene transcription, which results in the cell consuming less and obtaining more oxygen [9] (Fig. **1**). Specifically, HIF-1 boosts glycolysis and apoptosis, which lowers cell oxygen consumption *via* regulating the expression of glucose transporter type 1 (Glut1), B cell lymphoma-2 (Bcl-2). In addition, HIF-1 increases the production of red blood cells and small blood vessels to increase oxygen uptake by activating the expression of erythropoietin (EPO) and vascular endothelial growth factor (VEGF) [5, 9, 10]. Given the significant regulatory roles of HIF-1, some are attempting to target it to treat tumors (such as liver cancer, breast cancer, *etc*.) [11-13].

More than 500,000 new bladder cancer (BC) cases are diagnosed each year, and 200,000 deaths occur worldwide from this disease, making it one of the most prevalent cancers [14]. Although radical cystectomy treats BC, it fails to address micrometastases, causing tumor proliferation, angiogenesis, and metabolic changes, especially in muscle-invasive BC [15]. Hence, systematic treatment of surgery combined with drugs plays a key role. Surgery combined with targeted therapies such as fibroblast growth factor receptor and vascular endothelial growth factor receptor inhibitors have been used. However, the efficacy is uncertain due to only targeting angiogenesis or cell proliferation [16]. Exploring new, broad-acting targeted drugs is a priority.

Increasingly studies have found that HIF-1 has a systemic regulatory role in BC through complex signalling pathways [17, 18], which may provide a prior option in surgery combined with targeted therapy. Accordingly, we discussed the role of HIF-1 in glucose metabolism, metastasis, drug resistance, and other aspects of BC, and some possible inhibitors.

## 2 HIF-1 AND BC

### 2.1 HIF-1 and Glucose Metabolism

Glucose metabolism is the primary mode of cellular energy supply. Under normoxia, glucose is mainly catabolized through aerobic oxidative processes. Under hypoxia, it is decomposed primarily through glycolysis to provide energy to the cell [19]. However, it is incredible that tumor cells, whether aerobic or hypoxic, catabolize glucose mainly through glycolysis to provide energy rapidly [20]. Studies have confirmed that this rapid energy supply process is highly dependent on the enzymatic activity of glycolysis. Inactivation of the enzyme cuts off the energy supply and leads to the death of cancer cells [21]. Accordingly, some have explored the regulatory mechanisms of the expression of glycolytic enzymes and attempted to inhibit the activity of enzymes in various ways [22].

**Fig. (2):**
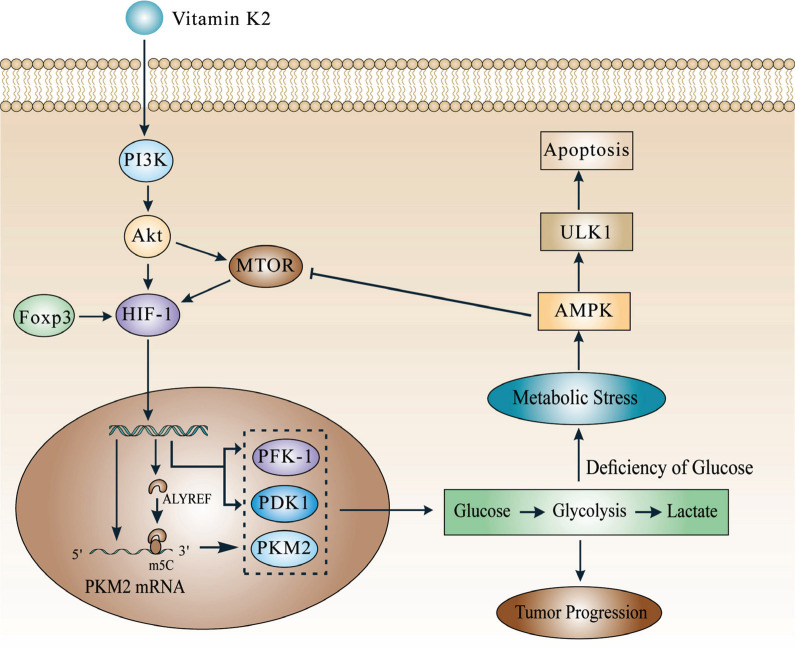
The role HIF-1 plays in glucose metabolism. HIF-1 accelerates glycolysis to create lactate by promoting the synthesis of glycolytic enzymes, which aids in the progression of BC. However, some factors (vitamin K2, Foxp3) increase level of HIF-1, which will lead to glucose deficiency and cell apoptosis.

Currently, it has been confirmed that HIF-1 is involved in regulating the expression of enzymes of glycolysis in BC cells [23, 24] (Fig. **2**). For example, the study through RNA immunoprecipitation assays found that after HIF-1 activates the transcription of pyruvate kinase muscle isozyme M2 (PKM2) (a key enzyme of glycolysis) to form PKM2 mRNA, the 3'-untranslated region (3'-UTR) of PKM2 mRNA will be modified by the 5-methylcytidine (m5C). The m5C site will then be recognized by the Aly/REF export factor (ALYREF) that is activated by HIF-1, which enhances the stability of PKM2 mRNA and leads to increased expression of PKM2 [25].

In addition, Zhang *et al*. [26] in BC cell lines SW780 and HT1376, through chromatin immunoprecipitation assays, found that HIF-1 promotes the expression of 6-phosphofructo-2-kinase/fructose-2,6-bisphosphatase 4 (PFKFB4). Subsequently, PFKFB4 promotes the synthesis of fructose-2, 6-bisphosphate (F2,6BP), which activates 6-phosphofructo-1-kinase (PFK-1) (a key enzyme in glycolysis). Similarly, Zhu *et al*. [27] found that HIF-1α may increase the expression of pyruvate dehydrogenase kinase 1 (PDK1) *via* the mammalian target of the rapamycin/ HIF-1α (mTOR/ HIF-1α) pathway.

When a large amount of enzyme is synthesized, glycolysis is enhanced and lactic acid is rapidly produced, directly or indirectly accelerating tumor progression through complex mechanisms such as suppression of tumor immunity and regulation of cellular signalling [25, 28, 29].

Interestingly, some found that despite the increased level of HIF-1α causing the synthesis of glycolytic enzymes, the end result was not cell survival but cell death (Fig. **2**). Specifically, the increased level of HIF-1α leads to excessive gluconic depletion or even glucose deficiency. BC cells will be in a state of metabolic stress, which promotes the activation of AMP-activated protein kinase/unc-51-like autophagy-activating kinase 1 (AMPK/ULK1) and inhibits the mechanistic target of rapamycin complex 1 (mTORC1), accelerating cell autophagy and thus leading to cell apoptosis [30].

At present, some factors that regulate the level of HIF-1α have been identified. For example, vitamin K2 promotes HIF-1α synthesis in BC cells *via* the phosphoinositide 3-kinase/protein kinase B/ HIF-1α (PI3K/Akt/HIF-1α) pathway [30]. In addition, Forkhead box P3 (Foxp3) prevents HIF-1α degradation and increases HIF-1α synthesis [24]. Then, HIF-1α promotes metabolic stress leading to cell death by AMPK/ULK1 or mTORC1 signalling [30].

### 2.2 HIF-1 and Angiogenesis

Angiogenesis is the process by which BC maintains its blood supply and is crucial for growth and pro-gression [31]. Evidence shows that angiogenesis is regulated by many factors, such as matrix metallo-proteinase (MMP) and interleukin-8 (IL-8) [32, 33]. Similarly, HIF-1α may also regulate angiogenesis. Specifically, Badr *et al*. [34] found that HIF-1α was positively correlated with microvessel density (MVD) in a pilot case-control study. Further studies revealed that HIF-1α can directly or indirectly promote the trans-cription of VEGF, thereby stimulating angiogenesis [35, 36]. Subsequently, the specific mechanisms revealed that activation of the PI3K/Akt/mTOR pathway increased HIF-1α synthesis and thus promoted VEGF expression [37], suggesting that HIF-1α mediates angiogenesis in BC *via* the PI3K/AKT/mTOR/HIF-1α/VEGF pathway.

Some drugs have been explored to inhibit angiogenesis by targeting HIF-1 [38, 39]. For example, low molecular weight fucoidan (LMWF) [40], acetone extract of *Angelica sinensis* (AE-AS) [39], and magnolol [41] inhibited the PI3K/AKT/mTOR pathway and thus HIF-1 in T24 cell or mouse model. In addition, AE-AS or magnolol can reduce ROS formation or prevent the inactivation of PHD and VHL, promoting the degra-dation of HIF-1α, thereby indirectly reducing angio-genesis [39, 41].

Surprisingly, Fus *et al*. [42] discovered that although HIF-1 expression was increased in low-grade BC, it was not raised in high-grade BC, which resulted in not promoting angiogenesis. We speculate that this is because the genome of high-grade BC is more prone to mutations, which may alter HIF-1 expression [43], but further evidence is required to confirm it.

### 2.3 HIF-1 and Proliferation

**Fig. (3):**
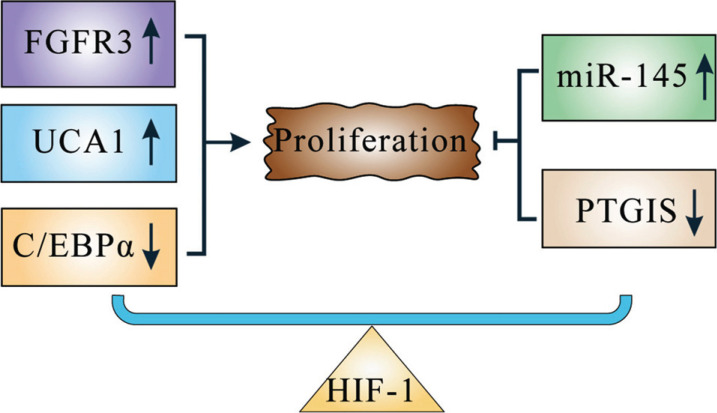
The role HIF-1 plays in the proliferation. HIF-1 either promotes or inhibits proliferation by regulating gene transcription.

How HIF-1 affects the proliferation of BC cells is extremely complex [44, 45] (Fig. **3**). On the one hand, some have found that HIF-1α promotes the proliferation of BC cells *in vitro*. For example, HIF-1α promotes the expression of fibroblast growth factor receptor 3 (FGFR3), which facilitates cell proliferation through the mitogen-activated protein kinase (MAPK) pathway [46]. In addition, HIF-1α promotes cell proli-feration by promoting the expression of uroepithelial carcinoma-associated 1 (UCA1) or repressing the expression of the transcription factor CCAAT/enhancer binding protein α (C/EBPα) [45, 47].

On the other side, the overexpression of HIF-1 suppresses the proliferation of BC *in vitro* (Fig. **3**). Specifically, when cold-inducible RNA binding protein (CIRBP) binds to the 3'-UTR of HIF-1α mRNA, the mRNA becomes more stable and promotes the expression of HIF-1α. Over-expressed HIF-1α then enters the nucleus, where HIF-1α methylates the HRE of the PTGIS gene, thereby preventing PTGIS transcription and tumor proliferation [44]. Moreover, HIF-1α upregulates downstream microRNA-145 (miR-145) and inhibits BC cell proliferation [48].

The above suggests that the role of HIF-1 in proliferation is dual. We speculate this is because of the changes in HIF-1 expression at different hypoxia levels [49]. Further studies are needed to reveal why HIF-1 expression differs at different levels of hypoxia and how it affects proliferation.

### 2.4 HIF-1 and Metastasis

Epithelial-mesenchymal transition (EMT) is a biological process in which epithelial cells are transformed into cells with a mesenchymal phenotype, and a key step is the reduction of intercellular adhesion [50]. The study confirms reduced intercellular adhesion in EMT is associated with tumor metastasis [51]. Specifically, the reduced adhesion function of epithelial calmodulin (E-cadherin), a transmembrane protein, or its reduced expression, leads to reduced intercellular adhesion, which promotes tumor metastasis [51, 52]. This suggests that EMT can promote tumor metastasis.

**Fig. (4):**
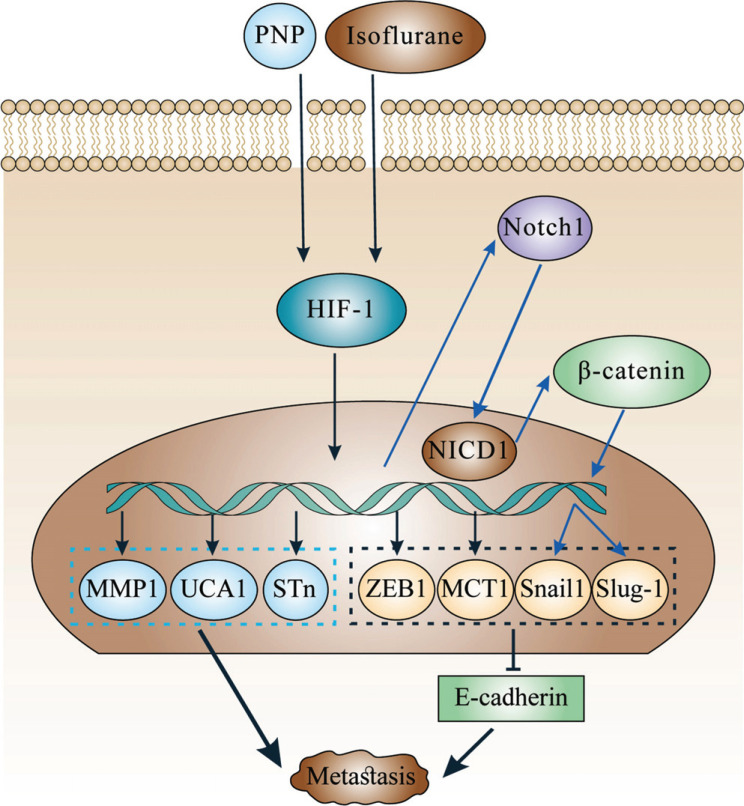
The role of HIF-1 in metastasis. Some factors (PNP, isoflurane) promote HIF-1 synthesis, thereby promoting gene transcription and BC metastasis.

Recently, HIF-1α was found to be involved in EMT of BC (Fig. **4**). Specifically, HIF-1α can promote the expression of zinc finger E-box-binding homeobox 1 (ZEB1) and monocarboxylate transporter isoform 1 (MCT1) in the animal model, which inhibits E-cadherin synthesis, thereby reducing intercellular adhesion and promoting EMT [23, 53].

Further studies have found that some drugs can affect HIF-1α, thereby promoting EMT (Fig. **4**). For instance, isoflurane or 4-nitrophenol (PNP) promotes the synthesis of HIF-1α and facilitates its entry into the nucleus, where HIF-1 induces the transfer of cytoplasmic notch receptor 1 (Notch1) to the nucleus. Then Notch1 in the nucleus promotes the nuclear transfer of cytoplasmic β-catenin, which subsequently promotes the expression of snail1 and slug-1, thereby inhibiting E-cadherin expression and reducing intercellular adhesion [51, 54, 55].

Moreover, HIF-1 promotes cancer metastasis in other ways besides EMT (Fig. **4**). For example, HIF-1α was found to promote the expression of sialyl-Tn (STn), MMP1, and UCA1, causing a weakened intercellular adhesion and thus promoting tumor metastasis [47, 56, 57].

### 2.5 HIF-1 and Drug Resistance

Therapeutic drugs for BC include chemotherapeutic drugs, immunotherapeutic drugs, and targeted drugs, which have shown certain efficacy and offer the possibility of effective treatment of BC [58]. But the subsequent discovery of drug resistance has also made therapy difficult [59]. Several studies have found that drug resistance is associated with reduced apoptosis and autophagy [60, 61]. Meanwhile, others found that HIF-1α may be related to drug resistance based on its role in regulating apoptosis and further explored its mechanism [18, 62].

Initially, HIF-1α was found to promote drug resistance to chemotherapy by regulating apoptosis (Table **1**). Specifically, Yu *et al*. [18] discovered that HIF-1α increased the expression of miR-424, which subsequently attached to the 3'-UTR of UNC-5 netrin receptor B (UNC5B) mRNA and Sirtuin4 (SIRT4) mRNA, thereby reducing the expression of them. When UNC5B and SIRT4 are reduced, it reduces death-associated protein kinase (DAPK) and enhances glutamine function to prevent apoptosis, thereby blocking cisplatin (CDDP)-induced apoptosis in T24 cell and mouse model.

Subsequently, others found that HIF-1α affects autophagy and thus inhibits apoptosis *in vitro* [63] (Table **1**). The specific mechanism is that HIF-1α promotes the expression of adenovirus E1B 19 kDa protein-interacting protein 3 (BNIP3), activating Beclin1. When Beclin1 is activated, it disrupts the B cell lymphoma/leukaemia-2 (Bcl-2)/Bcl-xL complex to promote autophagy, thereby eliminating CDDP-induced structural damage to cells and thus reducing apoptosis [64, 65]. This reveals that HIF-1α induces drug resistance to chemotherapy by promoting autophagy and/or reducing apoptosis, leading to drug resistance.

Interestingly, a more direct form of drug resistance is identified, in which HIF-1α affects the uptake and efflux of drugs by cancer cells (Table **1**). On the one hand, CDDP induces the accumulation of ROS, which increases the level of HIF-1α and thus promotes the expression of multidrug resistance protein 1 (MDR1), thereby leading to the excretion of drugs from BC cells [62]. On the other hand, HIF-1α may induce carbonic anhydrase IX (CAIX), which reduces drug uptake by regulating the activity of cell membrane ion channels and keeping the cells in an acidic state in a mouse model [66].

It is worth mentioning that immune escape has hindered the application of immunotherapy in BC [67], partly attributed to HIF-1 [24, 68] (Table **1**). For example, HIF-1α promoted VEGF expression, and then VEGF induced a decrease in CD8^+^ T cells and thus promoted immune escape [24]. In addition, the study suggested that HIF-1 may directly inhibit immune checkpoints (such as programmed cell death protein 1) and thus promote immune escape [68, 69].

### 2.6 HIF-1 and Treatment

Currently, given the extensive regulatory role of HIF-1 in BC cells, many inhibitors of HIF-1 have been explored [70, 71] (Table **2**). For example, SRT1720 [a kind of activator of Sirtuin 1 (SIRT1)] [70], Sulforaphane [71], artificial miRNA [72], magnesium isogly-cyrrhizinate (MI) [73], 16-Hydroxycleroda-3,13-dien-15,16-olide [74], 2-methoxyestradiol (2-ME) [75], and Solanum nigrum L [76] have been confirmed to inhibit HIF-1.

Among them, the mechanism of action of SRT1720 and MI is more straightforward. SRT1720 represses HIF-1α by activating SIRT1, deacetylating HIF-1α in the mouse model [70]. Moreover, MI activates miR-26b, which reduces the expression of NADPH oxidase 4 (Nox4), thereby inhibiting nuclear factor κB (NF-κB) entry into the nucleus and preventing NF-κB from promoting HIF-1α expression in the mouse model [73, 77]. Elucidating the mechanism of HIF-1 inhibitors provides a theoretical basis for further animal or human experiments.

## CONCLUSION

HIF-1 is a critical factor in BC adaptation to hypoxia and plays a crucial role in glucose metabolism, angiogenesis, proliferation, metastasis, and drug resistance of BC. Many inhibitors of HIF-1 have been identified, such as MI and SRT1720, but are not yet available for clinical use. The clinical efficacy of targeting HIF-1 needs to be evaluated. In addition, the mechanisms of action of HIF-1, such as the dual role of HIF-1 in high-grade BC, remain to be explored.

## Figures and Tables

**Table 1:** Role of HIF-1α in drug resistance.

**Gene**	**Function**	**Mechanism**	**References**
HIF-1α	inhibit apoptosis	HIF-1α/miR-424	[18]
HIF-1α	inhibit apoptosis	HIF-1α/BNIP3/Beclin1	[65, 66]
HIF-1α	promote drug efflux	HIF-1α/MDR1	[63]
HIF-1α	inhibit drug uptake	HIF-1α/CAIX	[67]
HIF-1α	promote immune escape	HIF-1α/VEGF	[24]

**Table 2:** HIF-1 inhibitors and mechanism of action.

**HIF-1 Inhibitors**	**Mechanism**	**References**
SRT1720	SIRT1/HIF-1α	[71]
Sulforaphane	HIF-1α/PHD	[72]
artificial miRNA	miRNA/ HIF-1α	[73]
MI	miR-26b/ Nox4/NF-κB	[74]
16-Hydroxycleroda-3,13-dien-15,16-olide	-	[75]
2-ME	-	[76]
Solanum nigrum L	NF-κB/HIF-1α or mTOR/ HIF-1α	[77]

## LIST OF ABBREVIATIONS

BC: Bladder cancer
HIF: Hypoxia-inducible factor
HRE: Hypoxia-response element
Bcl-2: B cell lymphoma-2
TME: Tumor microenvironment
